# Time for Awareness: The Influence of Temporal Properties of the Mask on Continuous Flash Suppression Effectiveness

**DOI:** 10.1371/journal.pone.0159206

**Published:** 2016-07-14

**Authors:** Weina Zhu, Jan Drewes, David Melcher

**Affiliations:** 1 School of Information Science, Yunnan University, Kunming, China; 2 Center for Mind/Brain Sciences (CIMeC), University of Trento, Rovereto, Italy; 3 Kunming Institute of Zoology, Chinese Academy of Sciences, Kunming, China; Monash University, AUSTRALIA

## Abstract

Visual processing is not instantaneous, but instead our conscious perception depends on the integration of sensory input over time. In the case of Continuous Flash Suppression (CFS), masks are flashed to one eye, suppressing awareness of stimuli presented to the other eye. One potential explanation of CFS is that it depends, at least in part, on the flashing mask continually interrupting visual processing before the stimulus reaches awareness. We investigated the temporal features of masks in two ways. First, we measured the suppression effectiveness of a wide range of masking frequencies (0-32Hz), using both complex (faces/houses) and simple (closed/open geometric shapes) stimuli. Second, we varied whether the different frequencies were interleaved within blocks or separated in homogenous blocks, in order to see if suppression was stronger or weaker when the frequency remained constant across trials. We found that break-through contrast differed dramatically between masking frequencies, with mask effectiveness following a skewed-normal curve peaking around 6Hz and little or no masking for low and high temporal frequencies. Peak frequency was similar for trial-randomized and block randomized conditions. In terms of type of stimulus, we found no significant difference in peak frequency between the stimulus groups (complex/simple, face/house, closed/open). These findings suggest that temporal factors play a critical role in perceptual awareness, perhaps due to interactions between mask frequency and the time frame of visual processing.

## Introduction

Although our subjective visual experience seems instantaneous and continuous, visual processing requires input to be combined over temporal windows ranging from tens of milliseconds [1], around 100–200 ms [2–4] or even hundreds of milliseconds in the cases of apparent motion [5,6] and object-substitution masking [7]. Integration windows are found also for audition [8–10], touch [11–14] and multisensory interactions [15,16] and thus may reflect a basic constraint on sensory processing (for review, see [17,18]). The temporal ordering of visual processing into temporal integration windows or “perceptual cycles” (for review: [18,19]) has implications also for the study of perceptual awareness. In the case of backward masking, for example, stimuli that are presented within the same integration window are combined together and observers cannot access the first target stimulus. The first, target stimulus is processed up to some point in the visual stream but fails to be distinguished from the mask in later stages of visual processing either due to integration masking or the influence of feedback processes (for review, see: [20]).

One of the main sources of evidence that stimuli that do not reach conscious awareness—and are thus “invisible”—are still processed to some degree by the visual system comes from studies using binocular rivalry [21,22] or Continuous Flash Suppression (CFS) [23]. Both the extraction of low-level visual features, such as orientation [24,25], spatial information [26] or motion [27], as well as the binding of low-level visual features based on Gestalt grouping cues, such as good continuation and proximity [28], have been reported to occur in the absence of awareness. It has also been reported that some effects generally attributed to “high-level” stages of visual processing may be possible without being aware of the percept, for example in face inversion [29–31], face expressions [32,33], semantic information [29,34,35] and information integration [36–39]. In particular, CFS has become the most popular tool for investigating visual processing outside of conscious awareness, although the exact nature of some of these effects remains controversial [40,41].

In a CFS paradigm, a series of contour-rich, high-contrast masks, called Mondrian patterns are continuously flashed to one eye at a steady rate (temporal frequency), causing a static low-contrast image presented to the other eye to be reliably suppressed throughout the entire viewing period [23,42]. In most of the studies using CFS, the temporal frequency for the mask images has been set to 10 Hz [23,42–47], while others have used 20Hz [32] or even 30Hz [48–50]. Some articles do not even report the frequency of masking used [51].

There is some preliminary evidence that temporal factors might play an important role in CFS. In the supplementary materials section of their seminal paper, Tsuchiya and Koch briefly reported a study of dominance durations for CFS masks of different durations [23]. This study used only a few subjects and measured duration of dominance, rather than the effectiveness of suppression as typically investigated in later CFS experiments. They reported weak effects for very long or short masks (and, hence, low and high temporal frequencies) but longer periods of suppression for mask durations between 80–320 ms (3.1–12.5 Hz). Another study compared CFS displays with either the high or the low temporal frequencies removed, and they found the high temporal frequency range was less effective at producing strong suppression [52]. In addition, one study comparing detection of flashed targets presented at different times during the sequence of CFS masks, either immediately after a new mask presentation, in the middle or right before a new mask refresh, found that suppression was weakest in the middle timing [53]. Moreover, detection of the flashed probe was most suppressed for mask temporal frequencies between 5–10 Hz. These studies suggest that the temporal frequency of the masks is an important factor, raising questions about the precise relationship between masking effectiveness and temporal masking frequency.

Given the role of temporal factors in many theories of visual awareness, such as phase coupling of neural oscillations across brain regions or re-entrant processing, we hypothesize that particular temporal frequencies of masking might yield the most effectiveness suppression in the CFS paradigm. In particular, previous studies have implicated alpha and theta rhythms in selective attention [54–58] and in the variability across trials in detecting near-threshold stimuli [59–61]. It has been suggested that these rhythms might reflect perceptual cycles, during which the visual system varies in its sensitivity to external stimuli [3,18,59,61,62].

Based on previous studies showing the importance of temporal factors to perception, the interaction of CFS with the time course of visual processing is expected to result in particular mask frequencies being more or less effective at suppressing the stimulus. Thus, the first aim of the present study was to systematically investigate the suppression effectiveness of a wide range of masking frequencies (0-32Hz).

It has also been shown that repetitive stimuli can entrain neural oscillations [63–67]. Given that the repetitive flashing of masks is an essential component of CFS, this raises the question of whether entrainment of rhythms corresponding to the masking frequency might influence perception. In order to investigate how long-term adaptation to the temporal frequency of the masking influences the depth of suppression, such as via repeated entrainment to one constant frequency, we also compared two different randomizations of the masking frequency. In one condition, trials with all different frequencies occurred in completely pseudo-randomized order; in the other condition, trials with the same masking frequency were grouped in blocks, and the order of the blocks was then randomized. Should the visual system be able to adapt to a given masking frequency, we would expect the required breakthrough contrast to be lower in the block-randomized condition. Alternatively, entrainment to a particular frequency might lead to stronger suppression as it might fine-tune the temporal effects of the mask.

Furthermore, in order to investigate whether the relationship between suppression effectiveness and masking frequency depends on the nature of the target stimuli, as might be expected from studies showing effects of target-mask similarity on backward masking [68,69] and binocular rivalry [70], both complex photographic stimuli (faces and houses) and simple geometric stimuli (closed/open shapes) were tested in this study. One possibility would be that the type of the stimulus would influence the depth of suppression [71–73] rather than temporal factors if the duration of the temporal integration window or perceptual cycle was similar for both types of stimuli. Alternatively, the use of very different stimulus types allowed us to see whether the most effective temporal frequency would also change depending on the stimulus.

More generally, the role of temporal factors in CFS is important for debates regarding the nature of “unconscious processing”. Many studies using CFS compare performance for detecting or discriminating stimuli that differ on a particular property, such as upright versus inverted faces [74,75]. One assumption implicit in these studies is that any feature which influences performance even for “invisible” stimuli must involve a specific computation that can occur even without awareness [76–78]. Given the hierarchical nature of visual processing, one potential explanation is that features that are processed relatively quickly are less disrupted by CFS, while conscious object formation, which may take up to 150–200 ms (for review: [18], is reset by the flash of a new stimulus. In support for this idea, detection under CFS improves for targets presented for longer durations, reaching plateau at around 100–150 ms [53]. Thus, one interpretation of “unconscious processing” of CFS is that it is most likely to occur for features that are processed quickly but less probable for stimulus features that require longer temporal integration periods or for processes that involve extensive feedback from higher-level areas. Multimodal integration has been shown to be able to affect break-through time in b-CFS paradigms [79], even though the underlying audio-visual integration stimulus lasted several seconds. However, in other cases no such effect was observed [80]. It is however not clear whether an audio-visual integration task can truly be seen as one long temporal integration, or instead rather as a sequence of many short visual presentations that are influenced by ongoing auditory stimulation. In any audio-visual task under CFS only the visual stimulus domain is being suppressed by the masking, allowing auditory information to influence the processing of incoming visual information. In addition, methodological issues with some of these studies have been raised, and future work will be necessary to give a clear explanation of audio-visual effects with CFS [40].

### Ethics statement

The experiment was approved by the ethics committee of the University of Trento, and performed according to the principles expressed in the Declaration of Helsinki. Written informed consent was obtained from all participants.

## Methods

### Subjects

A group of 15 subjects participated in the experiment (14 female, aged 19–29: mean = 23, SD = 2.9). The participants were students or postdoctoral fellows recruited from the University of Trento and were paid for their participation. All participants reported normal or corrected-to-normal vision and were naive to the purpose of the experiment.

### Apparatus

In the experiment, visual displays were presented on a 21 inch Mitsubishi CRT monitor (1024 × 768 pixel resolution, 160 Hz frame rate). Subjects viewed the presentation through a mirror stereoscope, with their heads stabilized by a chin-and-head rest. The viewing distance was 57 cm. To achieve good fusion of the display, the spatial distance between the left and right presentation areas as well as the angle of the mirrors were adjusted for each observer. Visual stimuli were presented in MATLAB (TheMathWorks, Inc., 2012) using the Psychophysics Toolbox [81].

### Stimuli and Procedure

**Fig 1:**
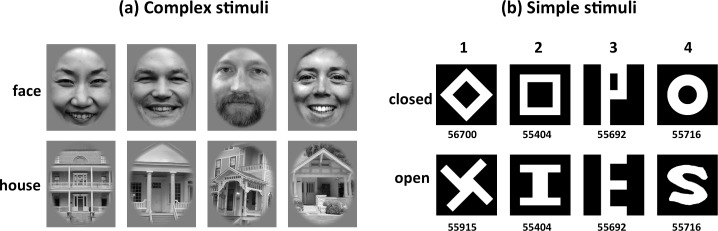
Sample images of (a) complex stimuli (faces and houses) and (b) simple stimuli (closed and open figures); the numbers below each simple stimulus represent the number of white pixels. There are 4 pairs of simple stimuli, and each pair was specially matched in their geometric properties (see text). To comply with PLOS copyright requirements, the original face stimuli were replaced with stand-ins that were preprocessed in an identical fashion and match the visual appearance of the actual stimuli.

Two sets of stimuli were prepared for the experiment. The first set were “complex” stimuli including 160 face images (one half of the face photographs were selected from the “Center for Vital Longevity Face Database” [82] and one half was randomly sampled from Google image search) and 160 house images. The images were converted from RGB color to gray-scale using MATLAB’s (TheMathWorks, Inc., 2012) built-in rgb2gray routine, which is a simple linear combination of the RGB color channels (0.2989*R + 0.5870*G + 0.1140*B). We equated the luminance histogram of the images by using the SHINE toolbox to minimize potential low-level confounds in our study [83]. Examples of the resulting stimulus images are shown in Fig 1(A). The stimulus images were cropped into a rectangular shape extending 7.9 × 9.9 degree, and displayed against a medium gray background.

The second set of stimuli, the “simple” stimuli included 4 pairs of one closed and one open geometric shapes each (inspired by Meng and her colleagues [73]). The areas (pixels) of white and black in each pair of stimuli were scaled to approximately the same (pair 1: 0.16% difference, pair 2, 3 and 4 identical). The first pair of stimuli consisted of identical diagonal line segments, and the second pair of stimuli had horizontal and vertical line segments. The only difference was the spatial configuration of these lines. The third pair of stimuli differed in the orientation and spatial configuration of one line segment. The fourth pair of stimuli was designed to have similar curvature. Stimuli are shown in Fig 1(B). While these stimuli may not be perfectly balanced regarding interocular conflict, they are considered to be well balanced regarding closure [73,84]. Stimuli were displayed against a black background. The stimulus images were cropped into a square shape extending 7.9 × 7.9 degree.

**Fig 2:**
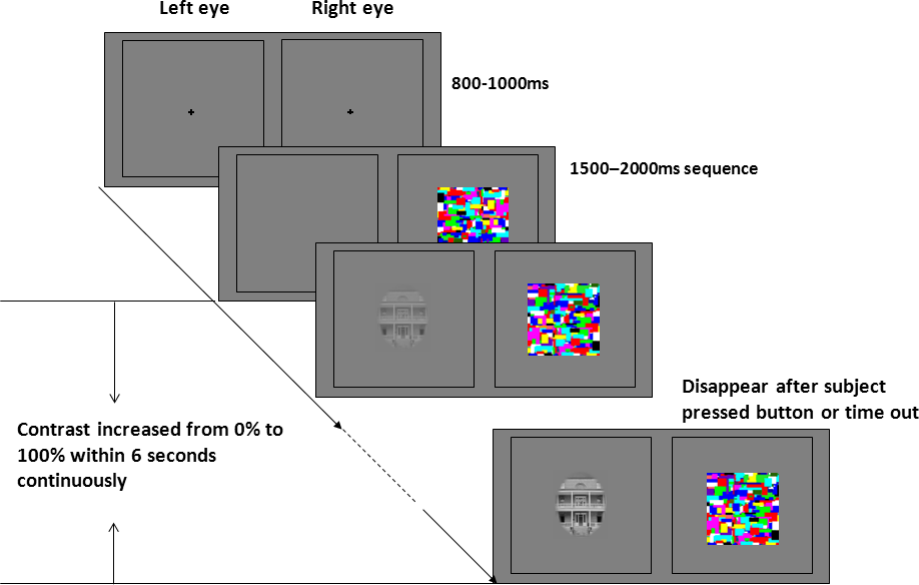
Schematic representation of the experimental paradigm: the contrast of the stimulus images increased continuously from 0% to 100% over a period of 6s. Subjects were instructed to press the space button as quickly as possible when they saw an image. Thus in our experiment, the breakthrough contrast was equal to the response time or duration of suppression.

High-contrast chromatic Mondrian CFS masks (very similar to those used in many other published CFS studies, e.g. [23]) were flashed to the dominant eye at predefined frequencies, while the stimulus image was presented to the other eye. The presentation size of the masks was 9.9×12.4 degree. Two static frames (11.9×14.7 degree) surrounded the outer border of stimuli and masks presented on the two sides of the screen, such that one frame was visible to each eye (see Fig 2).

Every subject completed 6 sessions comprised of 2 blocks each. Four of the sessions were complex stimuli and two of the sessions were simple stimuli. In the complex stimulus sessions, in each block 80 face trials, 80 house trials and 40 empty catch trials were shown. All of the images were presented in random order, and only once for each subject. In the simple stimulus sessions, in each block 80 target trials and 20 empty catch trials were shown. All of the images were presented in random order, and equally often.

Each trial started with a black central fixation cross (1.2×1.2 degree) shown for a random period between 800 and 1000ms. Masks were flashed to the dominant eye for a random period between 1500 and 2000ms before the stimulus began to fade in to the other eye. The contrast of the stimulus image increased from 0% to 100% in 6s continuously (around 0.1% per frame). Subjects were instructed to press the space button as quickly as possible when they saw any part of the target image, but not before they could see the target (breaking CFS, b-CFS). Subjects were informed that there could be catch trials, on which no response was to be given. Images disappeared after subjects pressed the button, or after the trial timed out (see Fig 2).

In the experiment, ten masking frequencies were used: 0, 1, 3, 5, 7, 10, 13, 16, 20 and 32Hz. There were two conditions to randomize the mask frequency during the experiment. In trial-randomized condition, trials with all different frequencies occurred in random order in all blocks. In block-randomized condition, trials with the same masking frequency were grouped in blocks, and the order of the blocks was randomized. Subjects were told that they could take a break anytime between trials if they so desired.

### Analysis

As a measure of central tendency, the mean of the breakthrough contrast was computed per subject on log-transformed data. In order to identify the optimal masking frequency at a finer scale, a skewed, raised Gaussian function was fitted to the measured breakthrough contrast (starting at 3Hz), and the location of the peak in the fitted function was determined. To test for statistically significant differences between peak frequencies, a bootstrap procedure was applied. Random sets of subjects were drawn (N = 14, with replacement), and the fitting procedure was then performed on the random set. In every repetition (N = 10000), the peak frequency of the resulting fit was calculated. Finally, the bootstrapped peak frequencies of the compared conditions were subtracted from each other, and a sign test was performed on the result. For other comparisons, as indicated, the break-through contrast (determined as the percentage of contrast at the time when the subject pressed the button) was analyzed by an ANOVA design for repeated measures with the masking frequency (10 different frequencies), randomization condition (trial-randomized vs. block-randomized condition) and stimulus type (complex vs. simple stimuli) as within-subject factors. Greenhouse–Geisser adjustments to the degrees of freedom were applied when appropriate.

## Results

**Fig 3:**
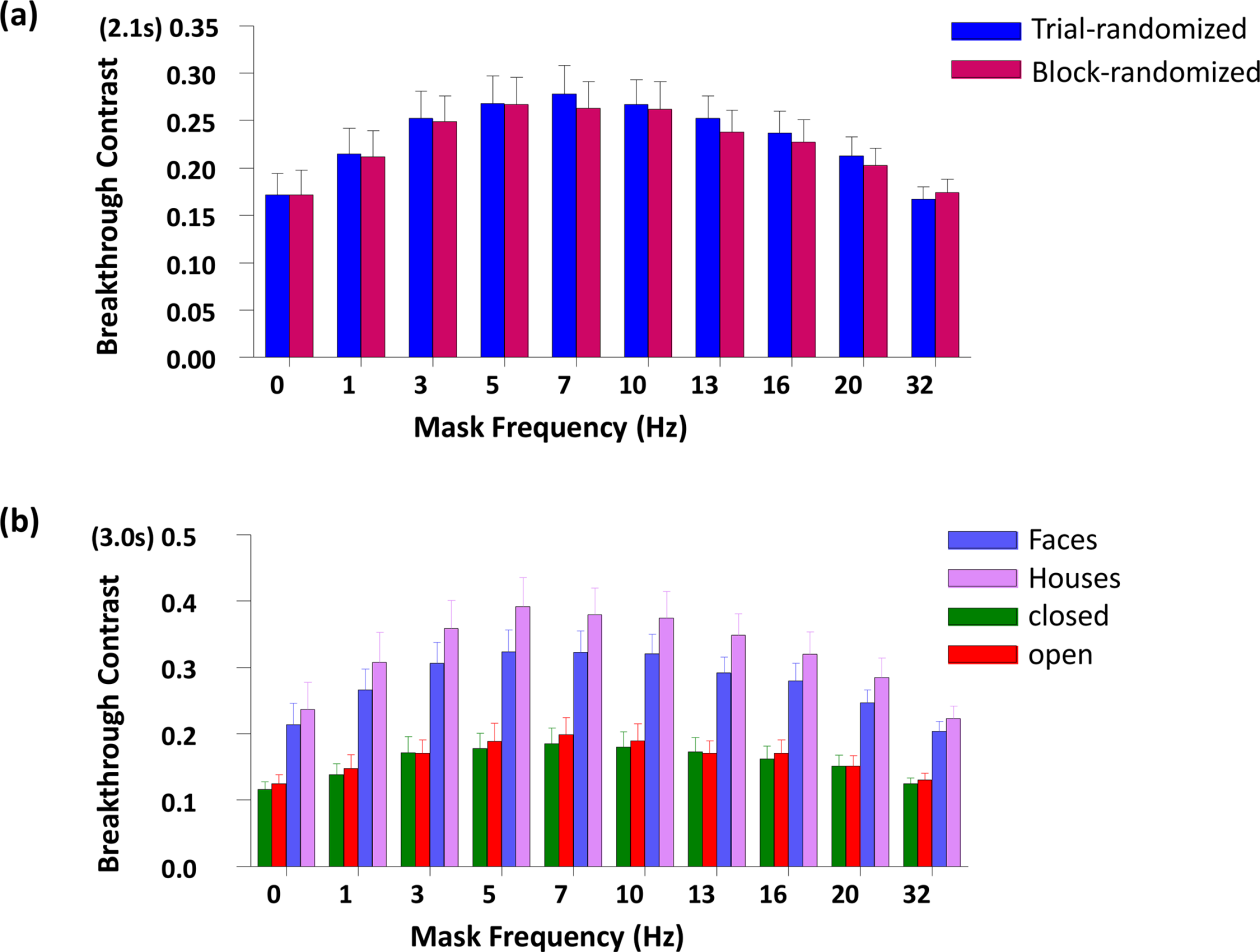
Breakthrough contrasts over ten frequencies. (a) for the two randomization conditions. (b) for the four stimulus groups.

The break-through contrast differed dramatically between temporal masking frequencies (F (9,117) = 16.182, p<0.001). As expected, the breakthrough contrast of the static mask (0 Hz: traditional binocular rivalry) was lower than all other higher frequency conditions (pairwise t-tests, df = 13, p = 0.040 or lower in all cases), except for the 32Hz condition, in which the breakthrough contrast was almost the same as the static mask (0Hz: 17.2%, 32 Hz: 17.1%, p = 0.938), see Fig 3. Subjects incorrectly responded to catch trials less than 4 of 80 trials on average (complex stimuli: 4 trials, simple stimuli: 2 trials), suggesting good adherence to the instructions.

We found a small but significant difference in breakthrough contrast between randomization conditions (trial-randomized: 23.2%, block-randomized: 22.7%; F(1,13) = 7.969, p = 0.014). However, there was no significant interaction between condition and frequency.

The complex stimuli yielded almost twice the average breakthrough contrast (29.8%) than the simple stimuli (16.1%) (F(1,13) = 97.26, p<0.001). For the complex stimuli, faces yielded smaller (27.8%) breakthrough contrast than houses (32.3%) (F(1,13) = 21.16, p<0.001). For the simple stimuli, closed figures yielded slightly but significantly smaller (15.8%) breakthrough contrast than open figures (16.5%) (F(1,13) = 9.657, p = 0.008).

**Fig 4:**
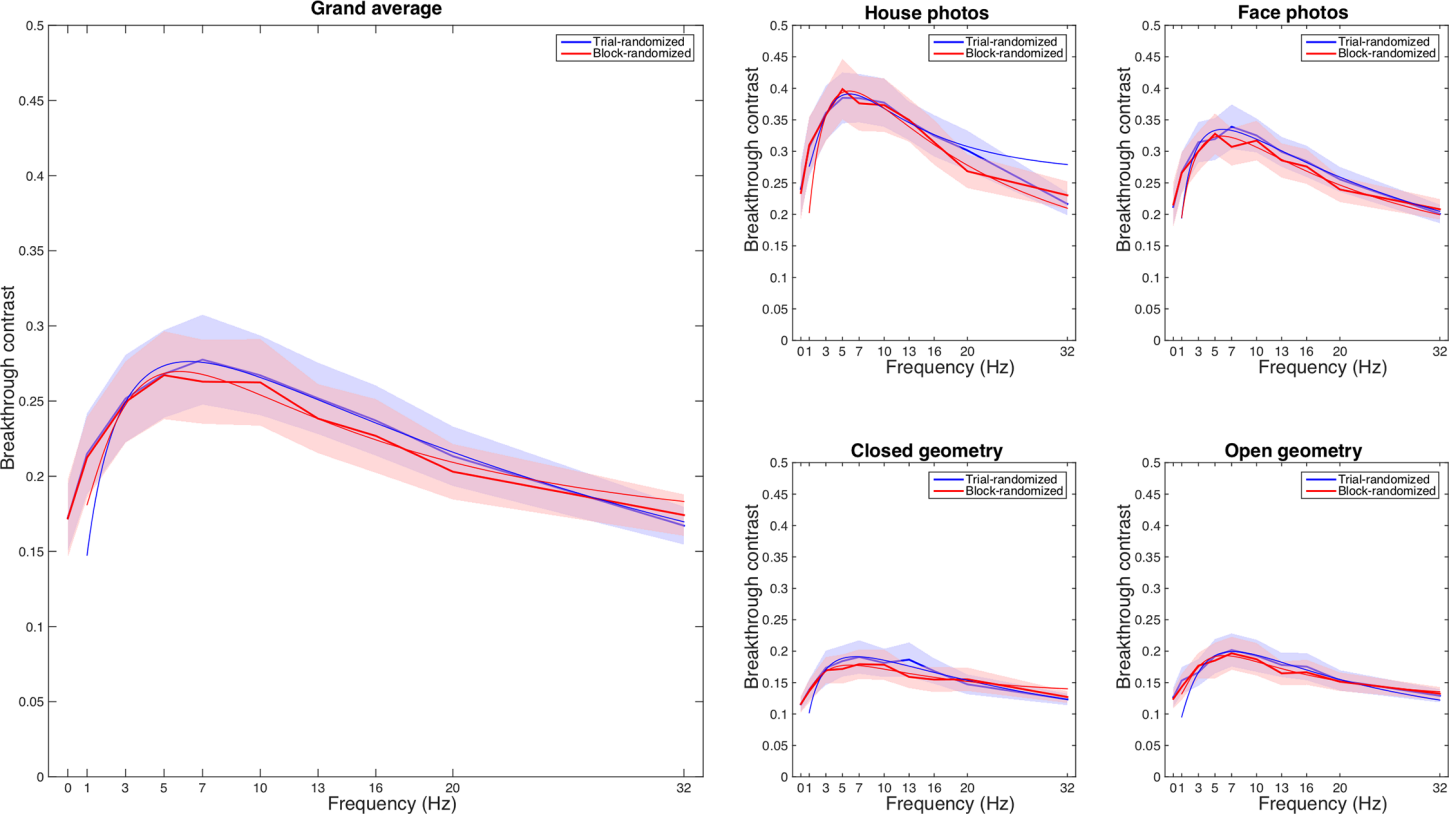
(a) Grand average and (b) different stimuli skewed-normal curves of breakthrough contrast on two randomization conditions: the bold curves represent the measured breakthrough contrast at given masking frequencies; the thin curves represent the result of fitting skewed-normal functions to the data.

**Fig 5:**
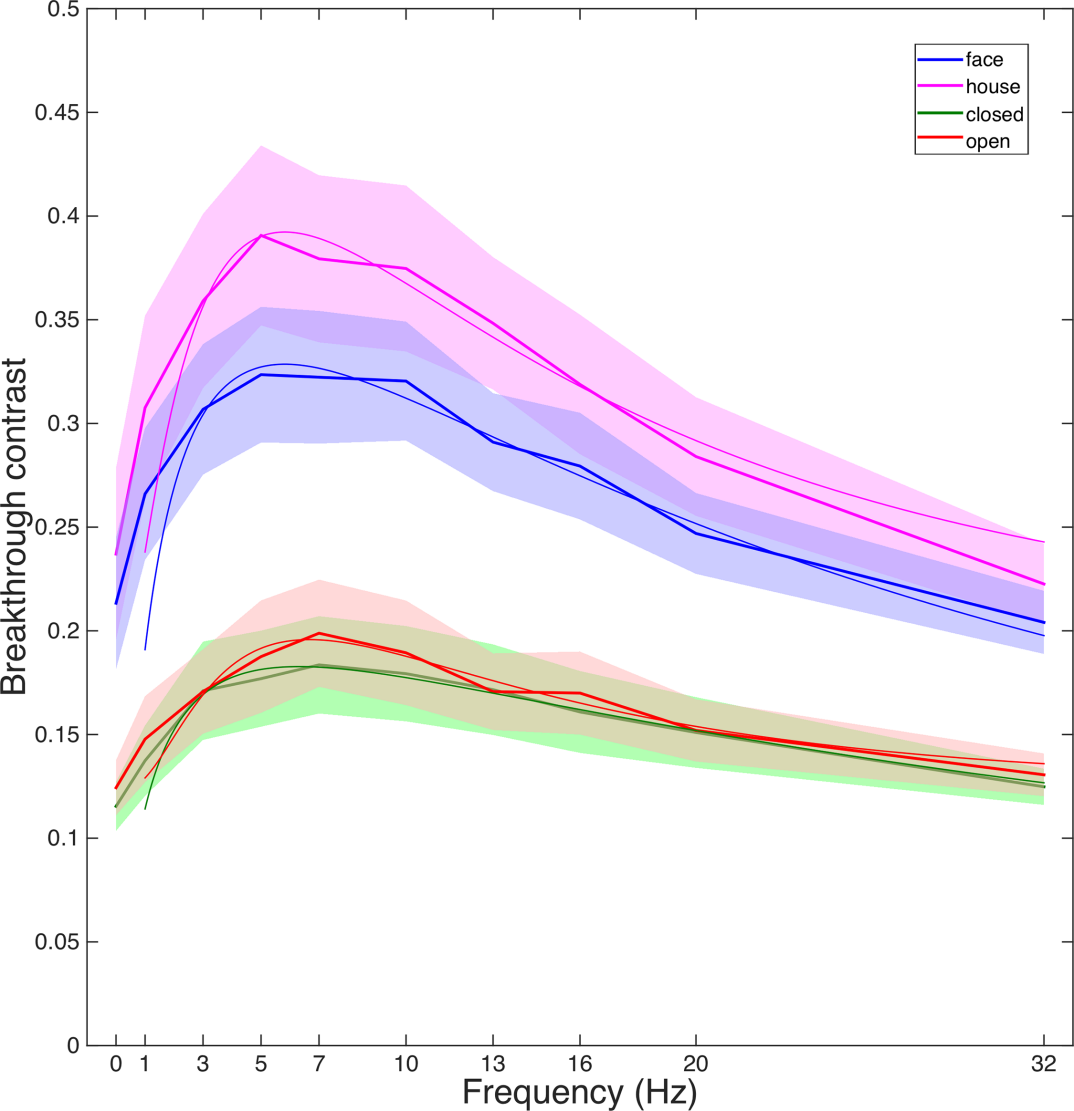
Skewed-normal curves of breakthrough contrast on 4 stimulus groups: the bold curves represent the average breakthrough time/contrast at a given masking frequency; the thin curves represent the result of fitting skewed-normal functions to the data.

In order to determine the location of the peak in breakthrough contrast more precisely, skewed-normal curves were fitted to the data. In Figs 4 and 5, the bold curves represent the average breakthrough time/contrast at a given masking frequency; the thin curves represent the result of fitting skewed-normal functions to the data. The peak frequency of the trial-randomized condition was slightly higher than the block-randomized condition (6.30Hz vs. 5.80Hz), however the difference was not significant (p = 0.4418). For both complex and simple stimuli, including face/house photos and closed/open geometry, the peak frequencies between trial-randomized and block-randomized conditions were not significantly different (complex: p = 0.7574, simple: p = 0.3778). Thus, peak frequency was remarkably consistent across conditions, always around 6 Hz and falling within 5–7 Hz (Table 1 and Fig 4).

**Table 1:** Peak frequency of two randomization conditions with different stimuli.

		Stimuli			
		Complex		Simple	
		Face	House	Close	Open
**Condition**	Trial-randomized	5.90Hz		6.95 Hz	
		5.90 Hz	5.85 Hz	6.65 Hz	7.15 Hz
	Block-randomized	5.80 Hz		6.05 Hz	
		5.80 Hz	5.80 Hz	5.90 Hz	6.10 Hz

As described above, the breakthrough contrast differed across target stimuli. Nonetheless, there was no significant difference in peak frequency between stimulus groups (complex/simple: 5.85Hz/6.55Hz, p = 0.5228; face/house: 5.95Hz/5.95Hz, p = 0.9458; closed/open: 6.30Hz/6.65Hz, p = 0.7388). Again, this comparison shows that the most effective frequency for CFS, across conditions, was around 6 Hz (Fig 5).

## Discussion

In this study, we investigated the temporal factors influencing the suppression effectiveness of CFS, measured as the break-through contrast of simple or complex target images. Because the contrast of the stimulus images increased from 0% to 100% continuously, the break-through contrast corresponded to the duration of stimulus suppression. Across conditions, the optimal frequency for CFS was around 6Hz. The most popular frequency choice in previous studies (10Hz) was slightly lower (4% absolute or 15% relative to 0Hz) in effectiveness. In practical terms, this finding is interesting because, while not very far from the optimum, the 10Hz masking frequency used in most current CFS studies may not be optimally effective. In fact, looking at the brief report included in the supplementary materials of the first CFS paper [23], which only included 3Hz, 12 Hz and one intermediate value in the range of interest here (around 6 Hz), the longest suppression durations were found at a value less than 10 Hz. This is also consistent with a later report showing that a high temporal frequency range, above 10 Hz, was less effective at producing strong suppression [52]. Likewise, detection of a briefly flashed probe was most suppressed when the temporal frequency of the CFS masks was between 5–10 Hz [53]. The current study goes further in more densely sampling different temporal frequencies in this range, allowing us to fit suppression effectiveness curves and identify a specific frequency value that was most effective and consistent across variations in stimulus type and for both regular and randomized temporal frequency blocks.

It is interesting to note that the most effective temporal masking frequency across the various conditions (6 Hz) was in the high-theta to low-alpha range. As described above, these frequencies have been implicated in detection of near-threshold stimuli (particularly oscillations in the alpha range [59–61], in oscillations in spatial attention (low alpha or high theta: [54,55,57,58]) and in re-current processing (theta: [3,18,62,85]). In particular, 6–7 Hz has been frequently implicated in several studies of the temporal frequency of attention [57,58,86,87]. According to “perceptual cycle” theories, the visual system samples incoming stimuli in discrete cycles in the theta range (150–200 ms) or alpha range (80–100 ms) [3,59,61,88]. In order to optimally disrupt this process prior to the stimulus being fully processed and made available for awareness, the temporal frequency of the mask should be slightly faster than the perceptual cycle. This would suggest that perceptual cycles operating in the theta range would be optimally disrupted with frequencies in the high-theta to low-alpha frequency range.

Another possibility is that CFS might suppress awareness of the target by repeatedly biasing competition towards the highly salient mask within the same level of visual processing, as in traditional binocular rivalry [70,89], prior to the completion of the processing of the target. Alternatively, the flashing of the mask might induce a phase reset [54,90] prior to completion of the perceptual cycle. A third possibility is that CFS acts by interfering with the process by which the completion of local processing of the target allows the information to be widely transmitted to other brain regions (e.g. Global Workspace Model: [91,92]). Further studies may shed light on why the influence of CFS is frequency dependent and how it disrupts the normal target processing that would lead to awareness.

To allow for comparison with traditional binocular rivalry, a “0Hz”-masking condition (a single static mask) was used in the experiment. As expected, the average breakthrough contrast of binocular rivalry (0Hz) was smaller than most of the other, higher masking frequencies with the exception of 32Hz, which resulted in almost the same breakthrough contrast as the binocular rivalry condition. In fact, the deeper, longer-lasting suppression generally aimed for when using Continuous Flash Suppression seemed to no longer be present in this condition. A possible explanation may be that high masking frequencies resulted in a degree of temporal fusion, which caused the visual system to process masks appearing at high frequencies in a similar way to static masking.

In the current study, the highest measured breakthrough contrast (at 5-7Hz) was only about 1.5 times bigger than that of our binocular rivalry condition, unlike previous reports in which there was a more than ten-fold increase in masking duration [23]. This apparent discrepancy in results may be somewhat reduced when considering a common minimum time for the stimulus to reach awareness: the contrast of our stimuli was increased gradually and comparatively slowly, starting at 0%. Because of this, break-through even in the absence of any masking would most likely not have been immediate since, even without distraction, target stimuli need to reach a certain minimum contrast to become visible. Subtracting this common minimum (not measured) would of course increase the relative difference between the 0Hz and 5-7Hz conditions. Another point is that the relation between mask strength and breakthrough time may not be linear, making further interpretation of the difference in masking duration between studies difficult.

We employed two modes of randomization of the frequencies used in this experiment, trial-wise and block-wise. These two modes were aimed at detecting changes in performance due to adaptation/habituation/entrainment to the masking frequency. Should the visual system be able to adapt to a given masking frequency across trials, we would expect the depth of suppression and in consequence the required breakthrough contrast to be lower in the block-randomized condition. In the opposite case, in the absence of adaptation, the resulting contrast levels between the different randomization conditions should be the same. The latter would also be true if adaptation happened on a short timescale, e.g. within each trial. Our results showed only a very small (even though statistically still significant) difference in breakthrough contrast between both modes. By fitting skewed normal curves to the data, we determined that the location of the peaks in breakthrough contrast along the frequency scale in these two randomization conditions were not significantly different from each other. A simple explanation for the small effects of randomizing frequency within blocks would be that subjects are more surprised or attentive (towards the masks) when the frequency of the masks changed unpredictably across trials.

To identify a possible influence of the interaction between the stimuli used and the effectiveness of different masking frequencies, we tested both simple shapes and more complex photographic stimuli. Not surprisingly, simple stimuli with well-defined contours yielded generally lower breakthrough contrast than complex photographic scenes. The complex stimuli used in this study were controlled for basic low-level properties (luminance and contrast), however they may have been confounded with other, uncontrolled factors. Still, consistent with previous studies, faces yielded lower breakthrough contrast than houses ([71,72] but [31]), and closed figures yielded lower breakthrough contrast than open figures [73]. Furthermore, the differences between stimulus groups were highly consistent (see Fig 3B). Critically, we found that there was no significant difference in peak frequency between any of the stimulus groups (complex/simple, face/house, closed/open), indicating that the effectiveness of temporal masking frequencies may be independent of the target stimulus category.

In summary, our findings support the idea that temporal factors play a critical role in perceptual awareness, not just in the case of masking [3,18,93,94] but also for CFS (see also: [53]. Our results suggest that the optimum masking frequency for CFS is relatively independent of the stimulus characteristics, with an optimum located in the high theta range. The absence of any target stimulus category effect on the optimal masking frequency suggests that the nature and timing of the masks, and their interaction with ongoing neural processes, may be the most critical factor in determining the optimal frequency for CFS masking. The finding that a CFS masking rate that is either too slow (less than 5 Hz) or too fast (over 20 Hz) loses its ability to suppress the target suggests that the temporal processing window between 50 ms– 200 ms is critical in the development of conscious access to a visual stimulus.
